# From the set-up of a screening program of breast cancer patients to the identification of the first BRCA mutation in the DR Congo

**DOI:** 10.1186/1471-2458-14-759

**Published:** 2014-07-28

**Authors:** Gertrude Luyeye Mvila, Sandra Postema, Guy Marchal, Erik Van Limbergen, Fons Verdonck, Gert Matthijs, Koen Devriendt, Genevieve Michils, Chantal Van Ongeval

**Affiliations:** Kinshasa General Hospital, Kasavubu University, University of Lubumbashi, Lubumbashi, DR Congo; Department of Radiology, UZ Leuven and Department of Imaging and Pathology, KU Leuven, Leuven, Belgium; Leuven University Centre for Cancer Prevention (LUCK), Leuven, Belgium; Faculty of Medicine, KU Leuven, Leuven, Belgium; Center for Human Genetics, UZ Leuven and Department of Human Genetics, KU Leuven, Leuven, Belgium

**Keywords:** Breast cancer, Central Africa, Prevention, Gene mutation, DRC

## Abstract

**Background:**

Breast cancer incidence in African population is low compared to western countries but the mortality rate is higher and the disease presents at a younger age and at a more advanced stage. The World Health Organisation and the Breast Health Global Initiative concluded that in low and middle income countries early breast cancer detection can be achieved by informing women on symptoms of breast cancer, on the practice of breast self-examination and clinical breast examination by trained health care workers. Based on these recommendations, we set up a breast cancer awareness campaign in Kinshasa, Democratic Republic of Congo (DRC). This paper describes the strategy that was established and the results that were achieved.

**Methods:**

A breast cancer awareness campaign was started in 2010 and data were collected until the end of 2012. Clinicians (expert group) trained nurses and health care workers (awareness groups) on clinical, technical and social aspects of breast cancer. Different channels were used to inform women about the campaign and clinical data (on medical and family history) were collected. The participating women were investigated with clinical breast examination by the awareness group. Women in whom a palpable mass was detected were referred to the hospital: they received a mammography and ultrasound and – in case of suspicious findings – additionally a core needle biopsy. In case of a positive family history, a blood sample was taken for genetic investigation.

**Results:**

In total, 4,315 women participated, resulting in 1,113 radiological breast examinations, performed in the General Hospital of Kinshasa of which 101 turned out to be malignant lesions. Fifty six percent of the women with breast cancer were less than 50 years old and 75% (65/87) were stage III tumors. A BRCA gene mutation was identified in a family with a severe history of breast cancer.

**Conclusions:**

Even without financial support, it was possible to start an awareness campaign for breast cancer in Kinshasa. This campaign increased the awareness on cancer of the women in Kinshasa. The results demonstrate that this campaign had an immediate impact on patients and their families.

## Background

An estimated total of 1,676,633 females were diagnosed with breast cancer globally in 2012 corresponding to an age standardized breast cancer incidence rate worldwide of 43.3 cases per 100,000 women and a mortality rate of 12.9 per 100,000, while in Europe the incidence rate of breast cancer is 94.2 per 100,000 and the mortality rate 22.4 per 100,000 [1]. In contrast with Europe the incidence rate in Sub-Saharan Africa is 25.5 per 100,000 and the mortality rate 19.3 per 100,000; for the Democratic Republic of Congo (DRC) the incidence rate is 23.5 per 100,000 and the mortality rate 14.2 per 100,000, showing a 4 times lower incidence but slightly lower mortality rate.

Considering population growth and aging, the incidence in breast cancer rates is rapidly increasing in Africa. In Uganda, for example, breast cancer incidence rate has doubled from 11 per 100,000 in 1961 to 22 per 100,000 in 1995 and increased from 18 per 100,000 to 31 per 100,000 in the period between 1991 and 2006 [2, 3].

Sylla et al. concluded that the incidence of cancer in Africa will increase tremendously solely due to demographic changes whilst that Africa is the least prepared continent to face this growth in cancer burden [4].

The recording of the occurrence of breast cancer is important to obtain a correct view on the prevalence of these cancers. However, few African countries have a cancer registry and the mature registries cover only a small population [1, 3, 5].

Despite the relatively low breast cancer rate among African women, the mortality rate is disproportionately high [6]. The reason of this high mortality rate is multifactorial: aside from the cultural aspect, namely the shortage of awareness among women and the lack of prioritization by the government, the lack of financial funding leads to a lack of diagnosis and treatment. Hence, many women are influenced by traditional healers and charlatans who have no clinical knowledge of malignant and benign breast pathologies [3–7].

Epidemiologically, the age at presentation is lower in Africa (mean age of 48 years) compared to Europe (mean age is 67 years in British white women) and breast cancer disease is more often stage III and IV [6, 8–10]. It is unclear whether the presentation at younger age and the more aggressive type of breast cancer in African women can be attributed to a genetic predisposition. Although some research was done in the African-American group, no scientific data exist on the prevalence of BRCA1 and BRCA2 gene mutations in Central Africa [8, 9].

As in many sub-Saharan countries, there is no national screening program for breast cancer in DRC [1].

The World Health Organisation (WHO) defines a national cancer control program as “a public health program designed to reduce the number of cancer cases and deaths and improve quality of life of cancer patients, through the systematic and equitable implementation of evidence-based strategies for prevention, early detection, diagnosis, treatment, and palliation, making the best use of available resources” [11].

However, a national cancer program based on a breast cancer screening test such as mammography is often not applicable in low income countries: the women are not aware of the pathology “cancer”, the medical equipment for the diagnosis of breast cancer is not available and there is no funding for the diagnostic or therapeutic interventions. The WHO reports that low and middle income countries (LMICs) have the option to implement early diagnosis programs based on awareness of signs and symptoms of breast cancer with prompt referral to diagnosis and treatment [12].

The Breast Health Global Initiative (BHGI) emphasizes the necessity of education for the set-up of an awareness program. This should be done by providers who first need to obtain the technical knowledge of breast self-examination (BSE) and clinical breast examination (CBE) while a basic level of knowledge of pathology and therapy should be available [13–16]. Bridges et al. described the different steps in order to come to a successful campaign on breast awareness and early detection more comprehensively [17].

This comprehensive ‘Framework for National Breast Cancer Control’ strategy consists of four themes: 1) building capacity, 2) developing evidence, 3) removing barriers and 4) promoting advocacy. Building capacity involves the training of nurses and relies on the creation of capacity for national public education. Concerning developing evidence, a description of the local etiology and the possibility of personalized treatment of breast cancer should be branched out. In the third theme, removing the barriers, insufficient access due to excessive costs for the patient to high patient related costs and the lack of access to the program of rural populations need to be solved. Finally, the fourth theme, promoting advocacy, focuses on survivors, quality of life, management of metastatic disease and support for leadership and staffing of advocacy groups.

Based on the information from the WHO, the BHGI and the paper of Bridges et al. it was concluded that health education messages must include the idea that breast cancer is curable in the majority of women provided that it is detected early, diagnosed accurately, and treated appropriately. To optimize successful outcome, communication methods need to be adapted to the cultural boundaries and taboos that invariably surround breast cancer, keeping in mind that those may differ among and within countries, depending on the social context and common healthcare systems [18].

The aim was to design a breast cancer awareness campaign in the DRC based on information (on early signs of breast cancer), education (on BSE and CBE) and diagnosis. The program was initiated by an interdisciplinary working group including oncologic surgeons, radiologists and nurses.

## Methods

The study has been approved by the ethics committee of the General Hospital of Kinshasa (GHK). All women who participated in this study have been informed about the procedures, filled out and signed the relevant questionnaire and informed consent forms.

### Strategy

**Figure 1:**
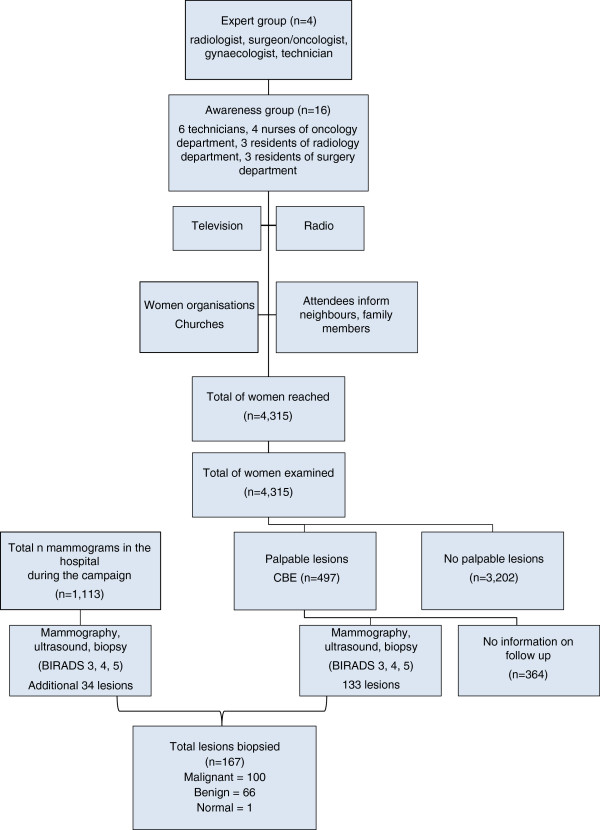
**Organisation of the breast cancer awareness campaign.**

Present study was realized in the department of medical imaging of the general reference hospital of Kinshasa. This is the largest hospital in DRC with a capacity of 2,500 beds and a medical staff of 186 physicians. The hospital has a tertiary function.The first step was the creation of an “expert group” possessing the required knowledge for the diagnosis and treatment of breast cancer and therefore able to provide a structure for the training of nurses and medical personnel. The expert group consisted of a radiologist, a surgeon/oncologist, a gynecologist and a technician. The physicians received training for at least 3 months at the KU Leuven Belgium (Figure 1). The expert group was in charge of the content of the education material and also for the composition of the awareness group who would do the teaching during the campaign.

The latter group included nurses and technicians who underwent a specific training in clinical examination of the breast, in auto-palpation and in providing information and education during the campaign [15]. A number of seminars were organized in the hospital for the physicians working in the radiology department, as well as for the nurses working in the oncology department in order to inform them on the different aspects of cancer in general, but also on how to diagnose and treat breast cancer. Finally the awareness group consisted of 6 technicians responsible for the mammographic examinations in the imaging department, 4 nurses from the oncology department, 3 residents from the radiology department and 3 residents from the oncology department. This group was complemented by two operated breast cancer patients who volunteered to participate in the campaign. The awareness group was split in two or three smaller groups of 5–6 persons, when several information sessions where planned on the same day. In these smaller groups, at least one medical doctor was present to answer more difficult medical questions.

### The recruitment procedure

The breast cancer awareness campaign was set up in Kinshasa in March 2010 and data were collected until the end of 2012. The channels to organize the awareness included television, radio, churches- and women organizations. To attain a large number of women we focused on religious meetings including conventional churches as well as the more recent popular Pentecostal churches which are mainly frequented by women. Before the start of the sessions, one person of the awareness group was appointed for the counting of the participants.

Each information session consisted of a general introduction on cancer, followed by a more detailed explanation on breast cancer. We focused on the importance of early detection, of all frequently occurring cancers in women but also on cancers in men such as prostate cancer. The posters contained images of cancer in both women and men. For women, the information focused on the necessity to consult a hospital for diagnosis and treatment rather than a center of witchdoctors.

Other posters described the method of auto palpation and the clinical examination performed by a health care worker. Detailed information on the treatment of breast cancer was provided to retain women from consulting traditional healers and other non-medical healers. The messages on the posters were kept simple and clear to also reach women with limited levels of education. The awareness group not only focused on meetings for women but also on groups in which men were participating. Some sessions were even organized for couples: in total one fourth to one fifth of the people attending the sessions were men. These men also asked questions related to cancer occurring in the male population.

Where possible the posters were replaced by slide presentations.

The meetings were announced on television and radio. During a presentation on television, the technique of mammography and ultrasound were explained and one of the patient explained about the organization and the goal of the sessions. On the radio the breast cancer awareness campaign was always presented by a radiologist who described the different early signs of breast cancer, the importance of breast self-examination and the difference between diagnosis and therapy in hospitals compared to witchcraft.

Each meeting started and ended with a question-answer session presided by a healthcare professional. After the information session, rudimentary examination rooms were set up and all participating woman were examined by a trained member of the awareness group.

The range of ages included in the study was from 18 years upwards. Male patients were excluded from present study.

Women with a palpable lesion were advised to consult the GHK, for further diagnostic work-up (mammography, ultrasound and biopsy). When a lesion was found, a core needle biopsy was performed.

Since most women could not afford an additional biopsy, only mammograms with the conclusion BIRADS 3, 4 and 5 were included in the study; the radiological examination with conclusion BIRADS 1 and 2 were excluded for further data collection and investigation [19]. For the TNM staging, only the clinical and radiological information could be used as the status of the lymph nodes was not always available and most of the surgical excised lesions were not all histologically investigated, therefore pathological staging was not possible [20, 21].

Regarding the clinical staging, the diameter of the tumor was recorded for all patients, and in order to compensate for the absence of pathological information, the radiological measurements were included in the staging. Due to the above mentioned problems, the description of the stages of the patients diagnosed in the DRC was different compared to the international TNM code (Table 1).

**Table 1:** **Adjusted clinical stages of breast cancer in DRC**

Clinical stage	Description
Stage 0	Absence of mass in the breast, but presence of a suspicious malignant lymph node
Stage I	A mass with a diameter less than 2 cm, without lymph nodes (documented on mammography or ultrasound)
Stage II	a mass with a diameter between 2 cm and 5 cm without lymph nodes (documented on mammography or ultrasound)
Stage III	A mass of more than 5 cm with the presentation of enlarged malignant lymph node clinically (documented on ultrasound)
Stage IV	The cancer shows an ulcerated mass with multiple lymph nodes that were pathologically enlarged and distant metastases*

*These patients received no additional biopsies but were immediately referred to the medical oncologist.

The collected data were analyzed in order to identify the variations in the etiology of the disease, but because of the organization of the study, no further conclusion on the incidence is made in this study. For all women diagnosed with breast cancer, family history was recorded and these family members were investigated. Blood samples were obtained for BRCA gene mutation screening.

### Genetic analysis

The genetic analysis was performed as described by Michils et al. [22]. During the data collection blood samples from a 29-year old patient with breast cancer, her affected mother and her aunt were taken for mutation analysis of the BRCA1 and BRCA2 genes.

## Results

**Figure 2:**
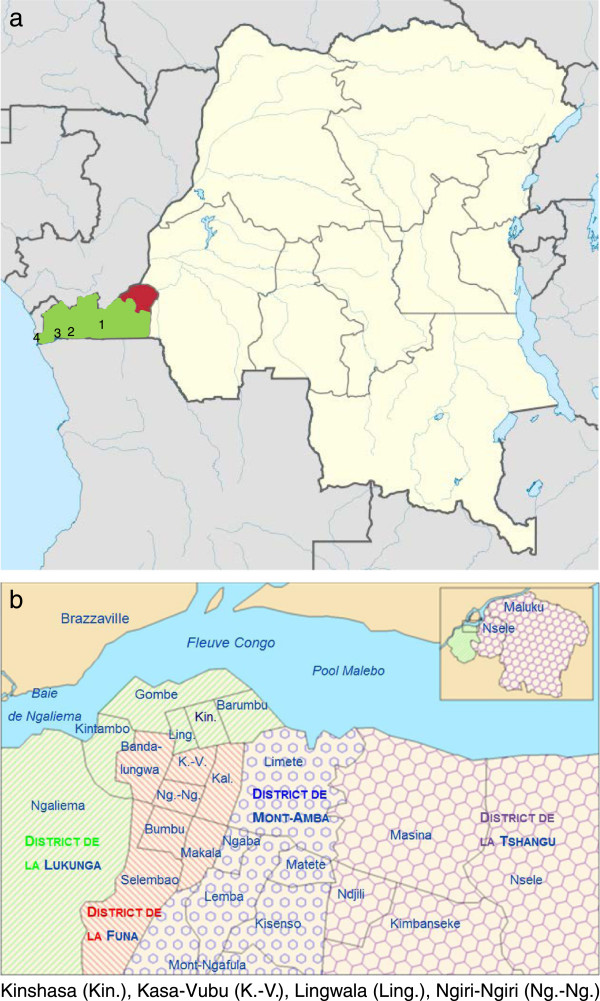
**Map of the Democratic Republic of Congo and of Kinshasa (capital).** 2**a**/Kinshasa (in red): 8,415,000 inhabitants, Bas-Congo (in green): 3,500,000 inhabitants with 1 = Kimpese, 2 = Matadi, 3 = Boma, 4 = Muanda. 2**b**/Kinshasa, capital with 24 communes.

**Figure 3:**
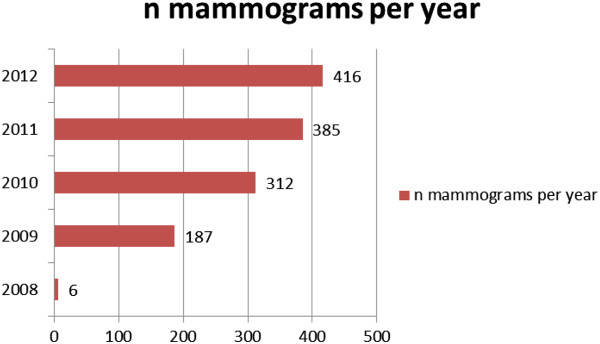
**Evolution of the number of mammograms (2009 till 2012).**

During the breast cancer awareness campaign a total of 64 women organizations and churches were visited: in the first year we visited 23 of them on our own initiative, in the second year, after having heard about our initiative, woman organizations and churches invited us for more information sessions. In the beginning, the campaign focused mainly on the capital Kinshasa with 8,415,000 inhabitants. In July 2012 we started to enlarge the reach of our campaign to the province of Bas-Congo (3,500,000 inhabitants) and we gave info sessions in Kimpese, Matadi, Boma and Muanda. The number of women visiting the campaign was 3,692. In July 2012 the awareness group reached another 623 women living along the motorway between Kinshasa and Muanda at the Atlantic Ocean (Figure 2a and 2b). The total number of women reached to date amounts to 4,315 and in 497 of these 4,315 women a palpable mass was found by the awareness group (CBE). Of these 497 women 133 consulted the radiology department of the GHK and were all diagnosed with BIRADS 3, 4, 5 lesions. Information on the 364 women with a palpable mass who did not present at the hospital was lost during follow up. Meanwhile, the breast examinations in the GHK increased from 312 in 2010 to 416 at the end of 2012, with a total of 1,113 mammography examinations in the studied period (Figure 3). Additionally, 34 lesions were diagnosed with BIRADS 3, 4, 5 in patients who were informed about this campaign by women out of the awareness group: a total of 167 lesions were biopsied [19]. In Table 2 demographic characteristics of women with BIRADS 3, 4 and 5 are summarized.

**Table 2:** **Socio-demographic characteristics of women diagnosed with BIRADS 3, 4, 5 (mammography, ultrasound) in the GHK in Kinshasa and Bas-Congo (DRC)**

Socio-demographic characteristics	n = 167	%
**Age (years)**		
Under 26	2	1.2
26-33	10	6
34-45	43	25.8
>45	112	67
Missing values	0	0
**Age at menarche (years)**		
11-13	41	24.5
14-15	111	66.5
16-18	15	9
>18	0	0
Missing values	0	0
**Education**		
Illiterate	0	0
Primary school	28	16.8
Secondary school	100	60
Higher	39	23.2
Missing values	0	0
**Marital status**		
Single	60	36
Married	75	45
Divorced/separated	3	1.8
Widowed	29	17.2
Missing values	0	0
**Occupation of the woman (profession)**		
Housewife	87	52
Manual labor	35	21
Medical doctor	2	1.2
Nurse	8	4.8
Student	1	0.6
Other	34	20.4
Missing values	0	0
**Number of children**		
0	41	24.5
1-3	53	31.8
4-5	42	25.1
>5	31	18.6
Missing values	0	0
**Family history of breast cancer**		
Yes	9	5.4
No	157	94
I don not know	1	0.6
Missing values	0	0
**Personal history of breast disease**		
Yes	17	10.2
Ovarian	4	2.4
Uterus	16	9.6
No	130	77.8
Missing values	0	0
**Religion**		
Christian	162	97
Muslim	0	0
Other/no religion	5	3
Missing values	0	0
**Motivation for consulting**		
Church	96	57.6
Family members	10	6
Radio	1	0.6
Television	3	1.8
Women organization	37	22
Other	20	12
Missing values	0	0
**3 years age standardized relative survival**		
Yes	137	82
No (died)	28	16.8
Missing values	2	1.2

n = total number of participants.

% = percentage of participants.

The other 946 patients were diagnosed as normal or benign and in order to minimalize the number of unnecessary procedures, no further biopsies were done.

**Figure 4:**
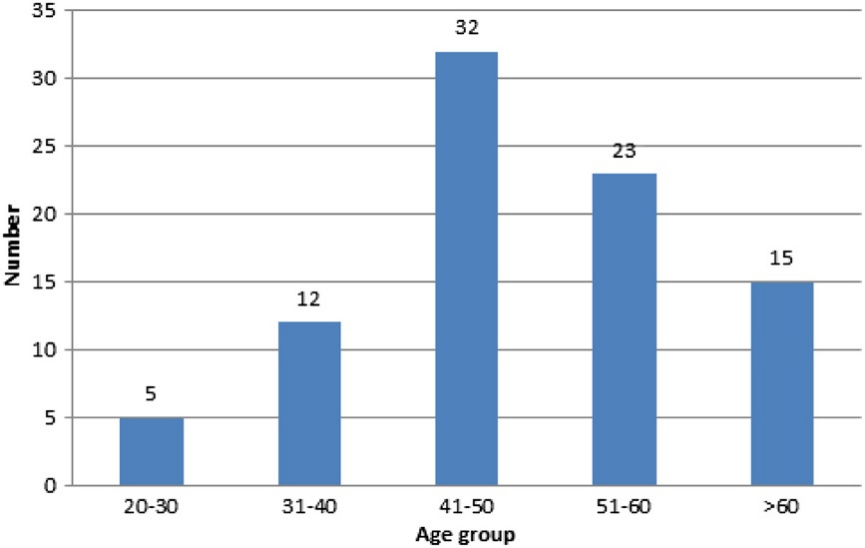
**Distribution of breast cancer for the different age categories.**

One malignant lesion was excluded from the whole study as only cytology was performed to diagnose the lesion. Of the 167 lesions which were correctly biopsied, 100 were found to be malignant, 66 were benign lesions, 1 was normal. Of the malignant lesions 87 (87%) were invasive tumors and 13 (13%) in situ carcinomas. The age distribution of these 87 women with malignant lesions was as follows: 5 (5.7%) were between 20 and 30 years old, 12 (13.8%) between 31 and 40 years old, 32 (36.8%) between 41 and 50 years old, 23 (26%) between 51 and 60 years old and 15 (17.7%) were more than 60 years old (Figure 4). The clinical stage distribution for the 87 invasive tumors was: 1 at stage 0 (1%), 2 at stage I (2%), 19 at stage II (22%), 65 at stage III (75%) (Table 3).

**Table 3:** **Clinical stage distribution of invasive breast carcinomas**

Clinical stage (n = 87)	n (%)
Stage 0	1 (1)
Stage I	2 (2)
Stage II	19 (22)
Stage III	65 (75)

### Genetic analysis

**Figure 5:**
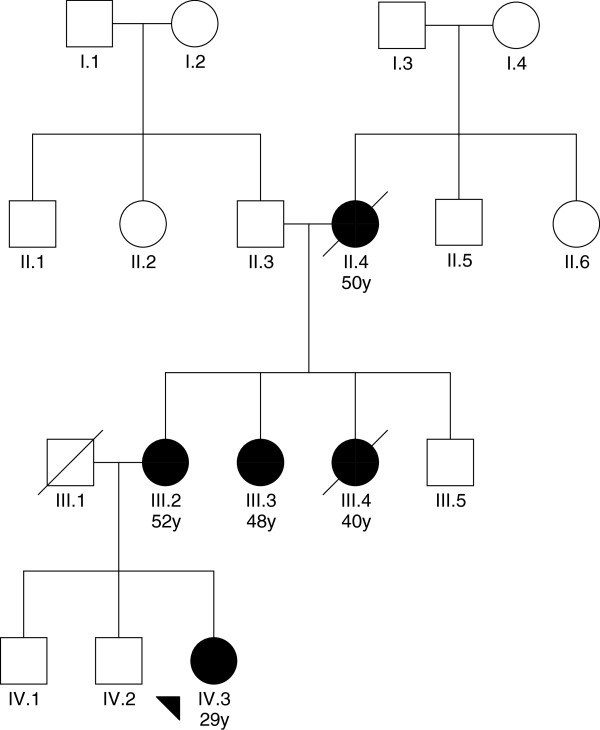
**Pedigree of the family that underwent BRCA1/2 genetic testing.** The age of disease presentation is indicated. The arrow refers to the index patient; blacked-in shapes indicate a breast cancer diagnosis.

One family with breast cancer at young ages was identified. Genetic analysis revealed the presence, in the heterozygous state, of the c.2389_2390 delGA mutation in the BRCA1 gene in all 3 members (Figure 5). The mutation leads to a frame shift at codon 797 of the BRCA1 gene (p.Glu797fs).

## Discussion

The incidence of cancer in developing countries is increasing mainly because of a population growth, a better control of infectious disease leading to an aging population and a better economic situation leading to a change of lifestyle [6]. Amir et al. reported a decrease in breast cancer incidence during the 1968–1996 AIDS epidemic registered by the Tanzanian Cancer Registry. However, once HIV was under control, women became older and cancer incidence was increasing [23]. In order to map the problem of breast cancer in women of Central Africa, a breast cancer control strategy based on breast cancer awareness, BSE and CBE was set up in the DRC. The implementation of this breast cancer control strategy resulted in an awareness campaign attended by more than 4,000 women in and around Kinshasa. As a result of the campaign, the number of women presenting at the hospital with a palpable lump increased significantly, while in the years before the campaign women were first seeking help in non-medical health services (witchdoctors). With more women presenting themselves with breast related problems at the hospital the expertise in breast diagnosis at the GHK improved and a better collaboration between clinicians and health care workers was achieved. In the department of imaging there was a significant increase in mammogram and ultrasound examinations. More than one thousand (1,113) radiological breast examinations were performed: 167 were concluded as BIRADS 3, 4, 5 and 100 cancers were detected (59% true positives). The detection rate in our study is higher compared to the results of Abruidis et al. [24]. In his study, in which an awareness program, for both men and women, was implemented in different villages in Soudan, 10,309 women were screened by CBE. Only 138 women were identified with breast abnormalities and referred to the hospital for further diagnosis. Of these 138 women, 118 were diagnosed: 101 with benign lesions, 8 with DCIS and 9 with invasive carcinoma (12% true positives). This difference can be attributed to the training of the health care workers in the awareness group of Kinshasa (better trained), the educational status of the women (rural versus non-rural situation) and to more practical issues (accessibility to the hospital). Information on cancer and BSE together with CBE resulted in a better detection of the early signs of breast cancer. This is in accordance with the findings of Mena et al. on the assessment of the impact of the Ghanaian non-governmental Breast Care International program on knowledge, attitudes and practices toward breast cancer in rural Ghana. In this paper the investigator concluded that the knowledge of the referent group on breast cancer appearing as a painless lump was only 53.3%, compared to the 82.3% of the intervention group, moreover the latter participants obtain significantly higher knowledge scores (odds ratio = 2.1, 95% confidence interval = 1.14-3.85) and achieve better BSE (odds ratio = 12.29, 95% CI: 5.31-28.48) [25].

Detailed analysis of our results showed that 56% of the cancers presented at an age between 30 and 50 years old. These results correlate with the data from Fregene et al., Basro et al. and Gukas et al. who stated that breast cancer incidence peaks between the ages of 35 and 45 years. This is approximately 10 to 15 years earlier than the peak incidence of western countries outside the western African region [6, 9, 26, 27]. Furthermore, 75% of the invasive tumors presented at stage III which is in agreement with studies conducted in Libya, Nigeria and Tanzania [28, 29].

We recognize that the present study has several limitations. A major one is that not all lesions seen on mammography or ultrasound were biopsied (only BIRADS 3, 4 and 5 lesions), nor a follow up of more than one year for the BIRADS 1 and 2 lesions was available. We realize that with this approach, some tumors might have been missed, but on the other hand - within the difficult financial context - we maximized the detection of the number of potentially treatable malignancies. However one consequence of this compromise is that the true incidence of breast cancer in the examined population is underestimated. Another limitation is the absence of an evaluation of the impact of the breast cancer awareness campaign on the knowledge of cancer and of the early signs of breast cancer in the participating women. But the increase of the number of women attending the sessions in churches and the increasing demand for new sessions suggest an improvement of the awareness in the population. Moreover, there is also a continuous and ongoing increase of women attending the mammography department.

In one family, a mutation was identified in the BRCA1 gene (c.2389_2390 delGA, p.Glu797fs). This mutation is predicted to lead to a truncated and non-functional protein and is therefore the cause of the disease in this patient and her family. This mutation is described in the Breast Cancer Information Core database and was initially identified in other patients of African origin [30]. Nevertheless, to the best of our knowledge, this is the first report of the diagnosis of a BRCA mutation carrier in DRC.

Despite the absence of any financial support for the campaign by the government, breast cancer awareness among the population increased and more women searched medical help. The goal of the campaign was also to inspire officials and local authorities to put breast cancer on the agenda of women’s health.

This campaign, even if it is only a start of a more widely introduced cancer campaign, will certainly have an impact on women’s health: Koon et al. demonstrated that advocacy and education, in particular through the efforts of breast cancer survivors and their partners, can play a crucial role in improving overall outcomes in developing countries [31].

## Conclusion

Introducing a breast cancer awareness campaign in the Democratic Republic of Congo, based on information by well-educated health care workers, breast self-examination and clinical breast examination, resulted in an increase of women looking for diagnosis and treatment in the hospital instead of consulting non-medical health care workers like witchdoctors. With an annual or bi-annual information day, knowledge on breast cancer can clearly reach out to more women and with some financial involvement of the government more women can be diagnosed at an earlier stage.
